# Supplementary material for: The causal role of circulating inflammatory markers in osteoporosis: a bidirectional Mendelian randomized study
Source: Front Immunol. 2024 Jul 18;15:1412298. doi: 10.3389/fimmu.2024.1412298 (PMC11291241; doi:10.3389/fimmu.2024.1412298)
Supplement: Supplementary file 1 [file DataSheet_1.zip › supplementary material/OSTEOPOROSIS-CIM/GCST90274793.tsv.gz_forest.pdf]

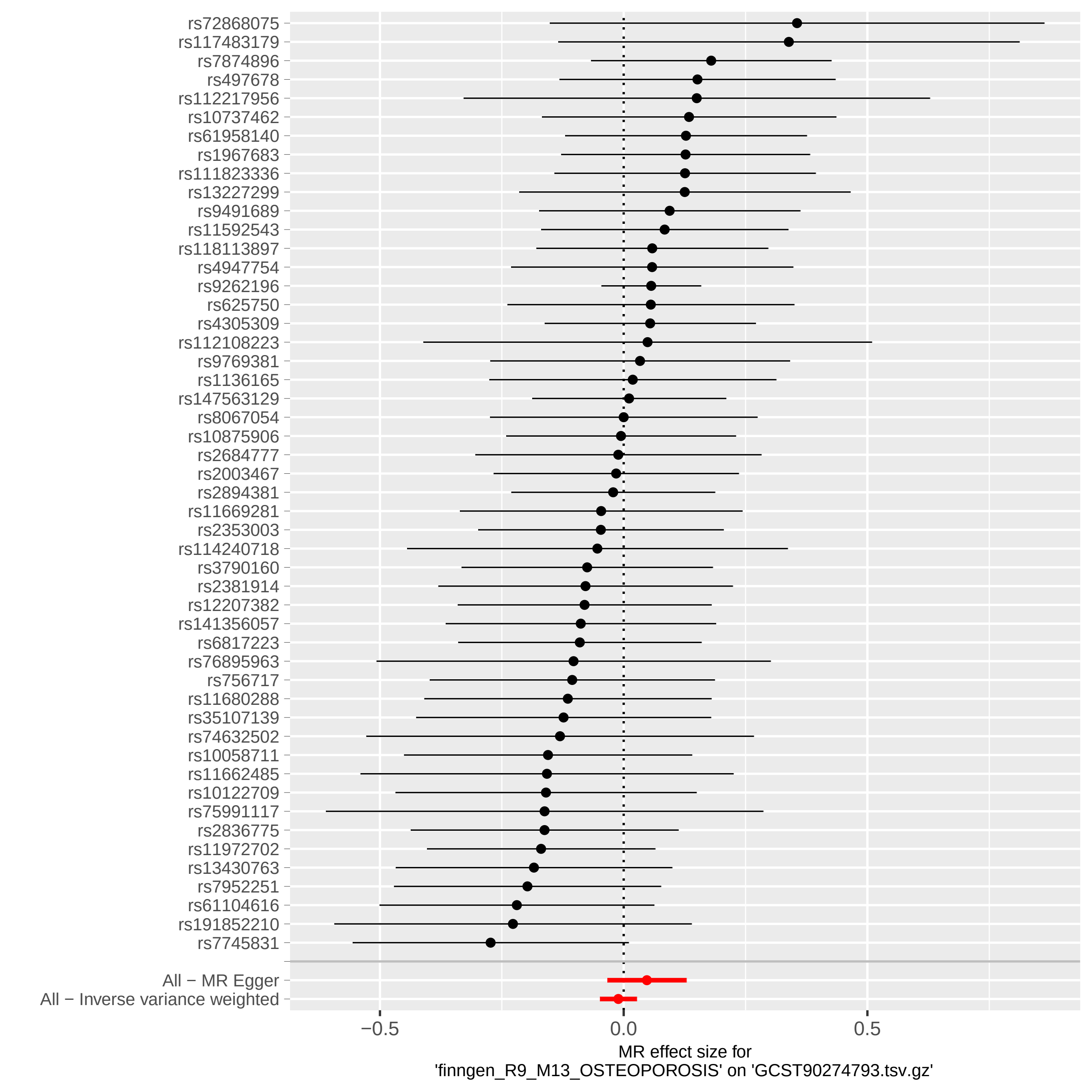
rs72868075
rs117483179
rs7874896
rs497678
rs112217956
rs10737462
rs61958140
rs1967683
rs111823336
rs13227299
rs9491689
rs11592543
rs118113897
rs4947754
rs9262196
rs625750
rs4305309
rs112108223
rs9769381
rs1136165
rs147563129
rs8067054
rs10875906
rs2684777
rs2003467
rs2894381
rs11669281
rs2353003
rs114240718
rs3790160
rs2381914
rs12207382
rs141356057
rs6817223
rs76895963
rs756717
rs11680288
rs35107139
rs74632502
rs10058711
rs11662485
rs10122709
rs75991117
rs2836775
rs11972702
rs13430763
rs7952251
rs61104616
rs191852210
rs7745831
All − MR Egger
All − Inverse variance weighted
−0.5
0.0
0.5
MR effect size for
'finngen_R9_M13_OSTEOPOROSIS' on 'GCST90274793.tsv.gz'
